# Multi-Organ Failure in Adult Patients Admitted to Intensive Care with Severe Burns—A Retrospective Cohort Study

**DOI:** 10.3390/ebj7030041

**Published:** 2026-08-04

**Authors:** William J. McIver, Randeep Mullhi, Elizabeth Chipp, Mansoor N. Bangash, Sarah E. Bache, Dhruv Parekh, Tomasz Torlinski

**Affiliations:** 1 West Midlands Burn Centre, Queen Elizabeth Hospital Birmingham, University Hospitals Birmingham NHS Foundation Trust, Mindelsohn Way, Edgbaston, Birmingham B15 2GW, UKtomasz.torlinski@uhb.nhs.uk (T.T.); 2 Institute of Inflammation and Ageing, University of Birmingham, Mindelsohn Way, Edgbaston, Birmingham B15 2WB, UK

**Keywords:** intensive care, multi-organ failure, multi-organ dysfunction syndrome, renal failure

## Abstract

**Highlights:**

**What are the main findings?**

- Multi-organ failure in severe burns follows a bimodal pattern, with sterile multi-organ failure in the first week being superseded by sepsis-related multi-organ failure in the latter period.
- Shock severity on admission and more aggressive initial fluid resuscitation are associated with the development of multi-organ failure, independently of burn severity.

**What are the implications of the main findings?**

- The changing risk factors for MOF over the time-course of ICU admission suggest that effective prevention strategies might differ over time, with early focus on haemodynamic optimisation and latterly a focus on sepsis surveillance and treatment.
- Early shock severity and fluid resuscitation volume represent potentially modifiable therapeutic targets that warrant testing in future prospective studies.

**Abstract:**

**Introduction:** Multi-organ failure (MOF) is the predominant cause of death in patients with burns who survive their initial injury. MOF can be “sterile” in nature (driven by the burn injury) or related to sepsis. The time course and predictors of MOF in major burns have not been well characterised. **Methods:** This is a retrospective cohort study of all adult patients with total body surface area (TBSA) burns > 15% admitted to the ICU of a UK tertiary burn centre between 2016 and 2025. Sequential Organ Failure Assessment (SOFA), DENVER2, and American Burn Association Sepsis Criteria were calculated on days 1, 3, 5, 7, 10, 14, 17, and 21 of ICU admission. Logistic regression models were used to explore variables associated with MOF. **Results:** In total, 131 patients were included. Patients who developed MOF were older, with more severe burns and inhalational injury. MOF incidence peaked at day 5 and then declined, while sepsis peaked at day 10. Renal failure and renal replacement therapy were uncommon, but both were strongly associated with reduced survival. Risk factors for early MOF were mainly related to burn severity, whereas sepsis was the predominant risk factor for late MOF. Shock severity on admission and more aggressive fluid resuscitation were also associated with MOF. **Conclusions:** Early organ dysfunction appears driven by the burn injury and associated shock, whereas later dysfunction is sepsis-driven. Renal failure is the organ failure most associated with mortality.

## 1. Introduction

Multi-organ failure (MOF) is the predominant cause of death in patients with burns who survive their initial injury [1,2]. MOF indicates dependency on advanced organ support therapies (e.g., vasopressors, mechanical ventilation, and RRT). It occurs at the severest end of multi-organ dysfunction syndrome (MODS), which is characterised by acute dysfunction in two or more organ systems, usually associated with an intense inflammatory insult [3,4]. American Burn Association (ABA) consensus guidelines recommend characterising MODS using a graded severity scale such as the Sequential Organ Failure Assessment (SOFA) score, only after acute resuscitation is complete (i.e., after day 3) [5]. Each additional organ failure is associated with a progressively worse prognosis in patients with burns [6,7].

Sepsis, in its most recent diagnostic criteria, is defined as life-threatening organ dysfunction caused by a dysregulated host response to infection [8]. Patients with major burns are predisposed to sepsis by multiple mechanisms, including loss of skin barrier to infection and the direct immunosuppressive effects of burn injury [9,10,11]. However, burn injury itself can also cause an inflammatory response great enough to trigger MOF, a phenomenon previously referred to as “sterile multi-organ failure” [12]. In addition, the hypermetabolic nature of major burns makes traditional markers of sepsis (e.g., pyrexia and tachycardia) unreliable in these patients [13]. Therefore, differentiating between sepsis and sterile MOF in burns is a common challenge to clinicians, and the relative contribution of the two to total organ failure burden is uncertain. Consensus guidelines provide some guidance but ultimately are based on a small volume of underlying empirical evidence [5,8].

The objectives of this exploratory study were as follows:

(1) To compare the characteristics of patients admitted to ICU with major burns who develop MOF with those who do not.
(2) To document the longitudinal progression of MOF in patients admitted to ICU with major burns.
(3) To investigate the association between individual organ failures and mortality.
(4) To explore the clinical risk factors for early and late MOF in severe burns.

## 2. Materials and Methods

### 2.1. Patient Selection and Clinical Management

Queen Elizabeth Hospital Birmingham (QEHB) is a large tertiary burn centre providing specialist adult burn care services for a population of roughly 13.7 million people covered by the Midland Burn Operational Delivery Network. Roughly 350 patients with burns are admitted every year. Patients requiring advanced resuscitation are admitted to the burn ICU, which provides specialist facilities for complex burn patients, including temperature-controlled rooms, in situ shower facilities and advanced organ support.

Consecutive patients meeting the following inclusion criteria between April 2016 and January 2025 were retrospectively identified:

- Age ≥ 16.
- Total Body Surface Area (TBSA) burn > 15%.
- Admitted to ICU at any point during their hospital stay.

Exclusion criteria were as follows:

- Admitted to ICU for palliative care only.
- Insufficient medical records to ascertain SOFA score on day 1 of admission or survival to hospital discharge.
- Transferred to another hospital whilst still requiring critical care.

Clinical management of all patients was undertaken according to standardised local protocols, in line with international standards [14]. Initial fluid resuscitation was with Hartmann’s solution as per Parkland’s formula [15]. Rate of fluid administration was subsequently titrated, aiming for urine output of 0.5–1 mL/kg/hr and mean arterial blood pressure > 65 mmHg. Human albumin solution (5% or 20%) boluses were administered according to clinician discretion, but local guidelines recommend withholding until at least 8 h post-injury. Local guidelines recommend measurement of residual gastric volumes (GRV) every 6 h during establishment of enteral nutrition, and addition of prokinetic agents (Metoclopramide and/or erythromycin) if GRV > 500 mL. Total parenteral nutrition (TPN) is prescribed on the advice of consultant nutrition specialists, usually when the patient cannot meet their nutrition demands enterally. Local guidelines recommend initiation of continuous renal replacement therapy (CRRT) in cases of severe metabolic acidosis, hyperkalaemia, pulmonary oedema or uraemic complications (encephalopathy, pericarditis) refractory to medical management. CRRT may also be used to treat hyperpyrexia in the setting of burns injury. Bronchoscopy was performed within 24 h of admission for all patients with suspicion of inhalational injury. Inhalational injury severity was reported using a simple bronchoscopic grading system [16]. “Mild inhalational injury” was treated as a separate entity to “No inhalational injury” for the purposes of our analyses.

### 2.2. Study Design

We conducted a retrospective analysis of data routinely collected as part of clinical care. Eligible patients were initially identified by querying a local burn injury database for all patients admitted to intensive care with burns during the study period. Clinical information was then obtained from the Prescribing Information and Communication System (“PICS”), which is the electronic health record in use at the intensive care unit of QEHB. Demographics, comorbidities, burn assessment and initial blood gas parameters were recorded from the time of admission. SOFA and DENVER2 scores were calculated for each patient on days 1, 3, 5, 7, 10, 14, 17 and 21 of ICU admission. The SOFA score is a MODS severity scale, commonly used to track organ dysfunction in sepsis [17]. It includes assessment of dysfunction in 6 organ systems—respiratory, central nervous system (CNS), cardiovascular, renal, liver and coagulation—giving a score of 0–4 for each system. As most of our patients were received intubated and sedated, it was not feasible to assess CNS function in a retrospective manner, and so this was excluded from the SOFA score, consistent with the approach taken by previous authors [18]. The DENVER2 score is a MODS severity score designed for use in major trauma, but also commonly reported in burns [19]. We therefore included it to aid comparability to previously reported studies. The DENVER2 score includes assessment of 4 organ systems: respiratory, renal, cardiovascular and liver, giving a score of 0–3 for each. The maximum cumulative daily score was retrospectively calculated for both MODS scores. At each time point, the metabolism/hyperglycaemia SOFA sub-score proposed by Boehm & Menke was also calculated [20]. The use of prokinetic agents or total parenteral nutrition, as a surrogate for feeding intolerance and gut failure, was recorded. The presence or absence of sepsis was also recorded according to ABA criteria [5]. Briefly, this required acute physiological disturbance in combination with a positive microbiological culture or pathological tissue source. Review of physiological parameters, culture results and ward round documentation (including daily input from infectious disease specialists) was used to retrospectively diagnose sepsis at each time point. Clinical outcomes, including total length of stay, length of ICU stay, ventilated days and survival to hospital discharge were recorded from the electronic health record.

Patients were divided into 3 groups for further comparison. The “early death” group was defined as actively treated patients who died within 3 days of admission (during and immediately post the resuscitation phase). The “MOF” group was defined as patients with a total SOFA score ≥ 6 at any point after day 3. This cutoff was chosen based on previous studies [6,21]. The “no MOF” group was defined as patients with a total SOFA score < 6 at all available time points after day 3. Individual organ failure was defined as an organ-specific SOFA score ≥ 3 at any point after day 3 [22,23]. Late MOF was defined as total SOFA score ≥ 6 after day 7, and early MOF was defined as total SOFA score ≥ 6 prior to day 7. Frailty was defined as a clinical frailty scale (CFS) ≥ 4 prior to burn injury [24]. The Charlson comorbidity index was calculated for each patient. This score combines age with the presence/absence of 16 comorbidities and was used in this study to represent the burden of chronic disease [25]. The Baux score is a risk score known to predict mortality in burns, calculated as age + TBSA. The revised Baux score (r-Baux) also considers the presence of inhalational injury [26,27]. Acute physiology and chronic health evaluation (APACHE II) score was calculated at the point of ICU admission [28]. Where a pre-intubation Glasgow coma scale (GCS) was not reliably recorded, this was assumed to be 15 for the sake of APACHE II scoring.

### 2.3. Statistical Analysis

Categorical data were presented as n (%) and analysed using Fisher’s exact test to compare 2 groups and the Chi-squared test to compare 3 groups. Continuous data are presented as median (interquartile range) and were analysed using the Mann–Whitney U test for comparison of 2 groups and the Kruskal–Wallis ranked sum test for comparison of 3 groups. Logistic regression models were used to test for associations between the Charlson comorbidity scale/TBSA/inhalational injury/sepsis and the occurrence of early and late MOF. The independent variables were chosen based on known risk factors for poor outcomes in burns [2]. Variance inflation factor (VIF) was used to test for multi-collinearity, and variables were removed if VIF > 5. Univariate logistic regression was used to test for associations between markers of shock/severity of illness on admission and occurrence of MOF. Results were considered statistically significant if *p* < 0.05. All statistical analyses were performed using Microsoft Excel (version 2506) and R (version 4.5.1).

## 3. Results

### 3.1. Patient Characteristics

**Figure 1:**
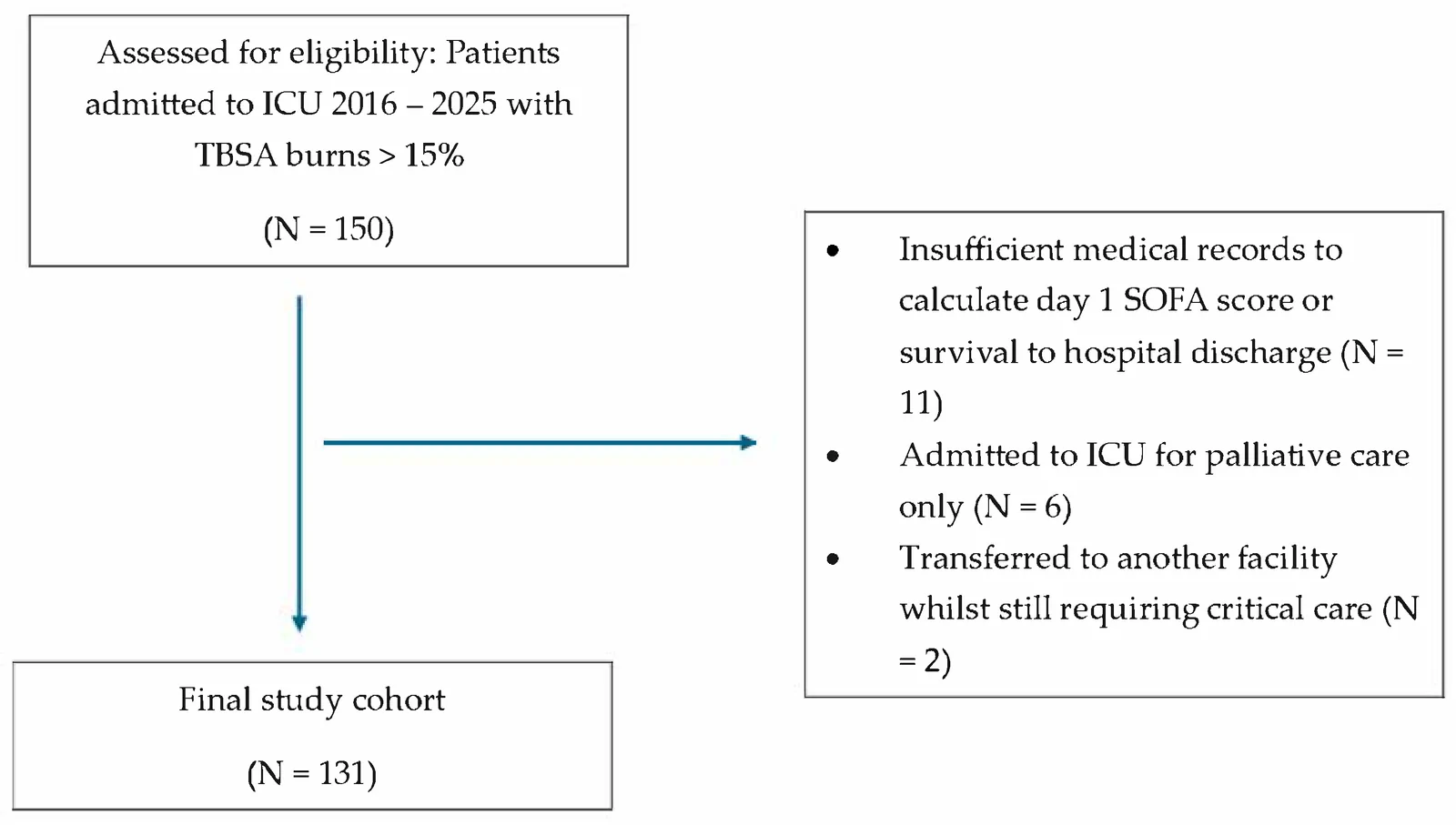
Consort diagram representing process of screening and enrolment into study and reasons for exclusion. ICU = intensive care Unit. TBSA = total body surface area.

As shown in Figure 1, in total, 150 patients were screened. In total, 131 patients met the inclusion criteria and were enrolled in the final study cohort.

Median length of ICU stay was 14 days (6–27). In total, 121/131 (92%) of patients were intubated within 24 h of injury, including 55/131 (46%) prehospital, 36/131 (30%) in the emergency department and 23/131 (19%) in theatre. During their ICU admission, 60/131 (48%) received a tracheostomy. The overall group characteristics, as well as comparison between groups, are shown in Table 1. In total, 18/131 (14%) patients died within 3 days of admission, 78/131 (59%) survived the first 3 days but later went on to develop MOF, and 35/131 (27%) patients survived the first 3 days and did not develop MOF. Amongst the early death group, 15/18 (83%) of patients died following compassionate withdrawal of life-sustaining therapies. Patients in the early death group were older, were more likely to be frail, were more likely to smoke, had more severe burns and had more severe inhalational injuries than the other groups. Patients who developed MOF were older, with more severe burns and more severe inhalational injuries than patients who did not develop MOF. Clinical outcomes (length of stay, ventilator-free days, and number of theatre trips), except survival to discharge, were all significantly worse in patients who developed MOF compared to those who did not. There were no significant differences in sex, race, BMI, involvement of head/neck or requirement for escharotomy between the groups.

### 3.2. Progression of Multi-Organ Failure

**Figure 2:**
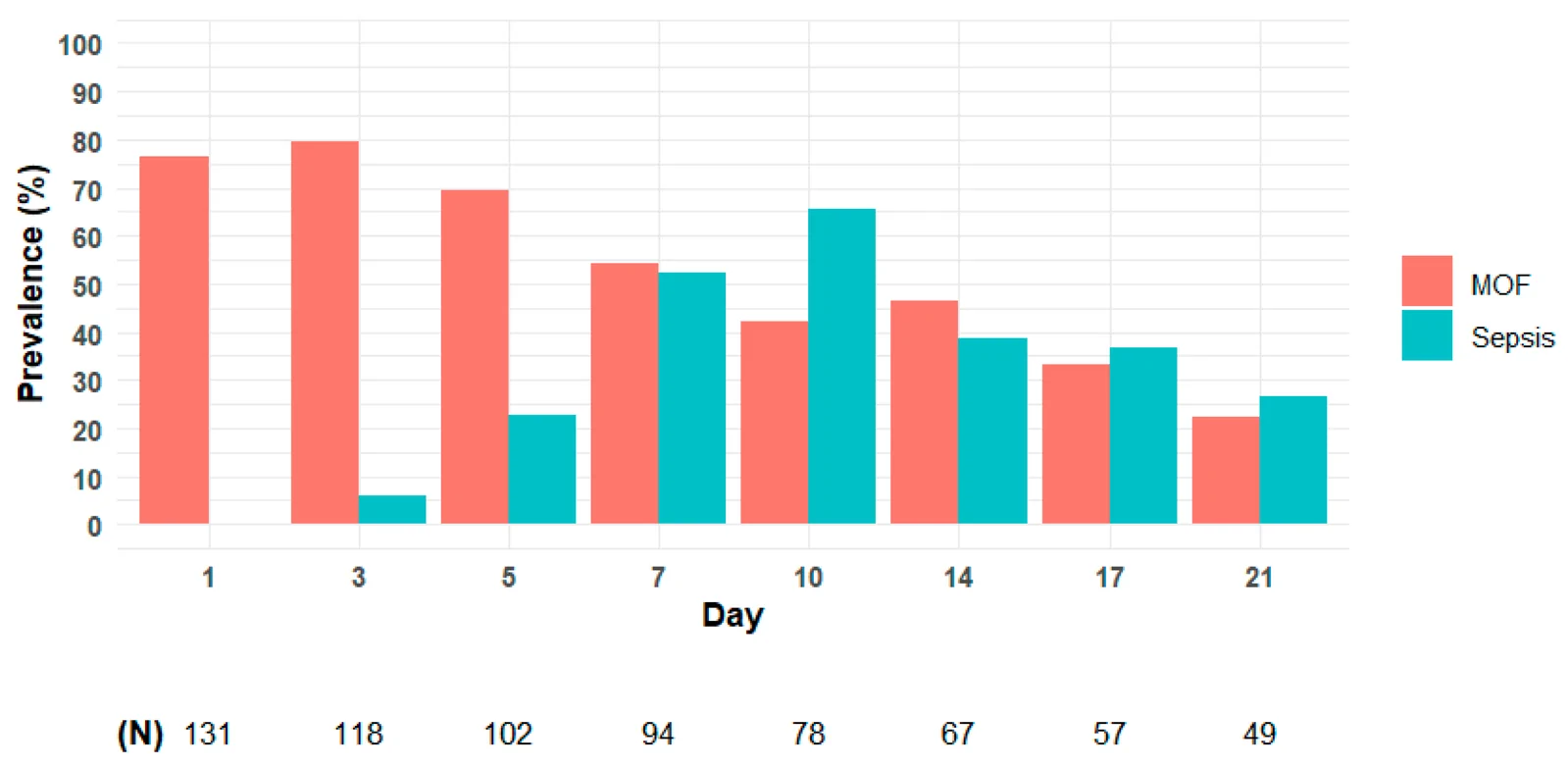
Bar chart showing prevalence of MOF and sepsis by day of intensive care unit admission. (N) represents number of patients alive and on ITU at each time point. MOF = multi-organ failure.

Figure 2 shows the prevalence of sepsis and MOF at each time point. Overall, 80/131 (61%) of patients developed sepsis at some point during their ICU admission. Sepsis prevalence increased to a peak of 51/78 (65%) of patients at day 10, after which it declined. MOF prevalence generally declined throughout ICU admission, from a peak of 71/102 (70%) on day 5 to 11/49 (22%) on day 21. Although ABA criteria recommend against characterising a patient as suffering from MOF until day 3, we have included the prevalence at these time points to represent acute organ dysfunction in the resuscitation phase.

**Figure 3:**
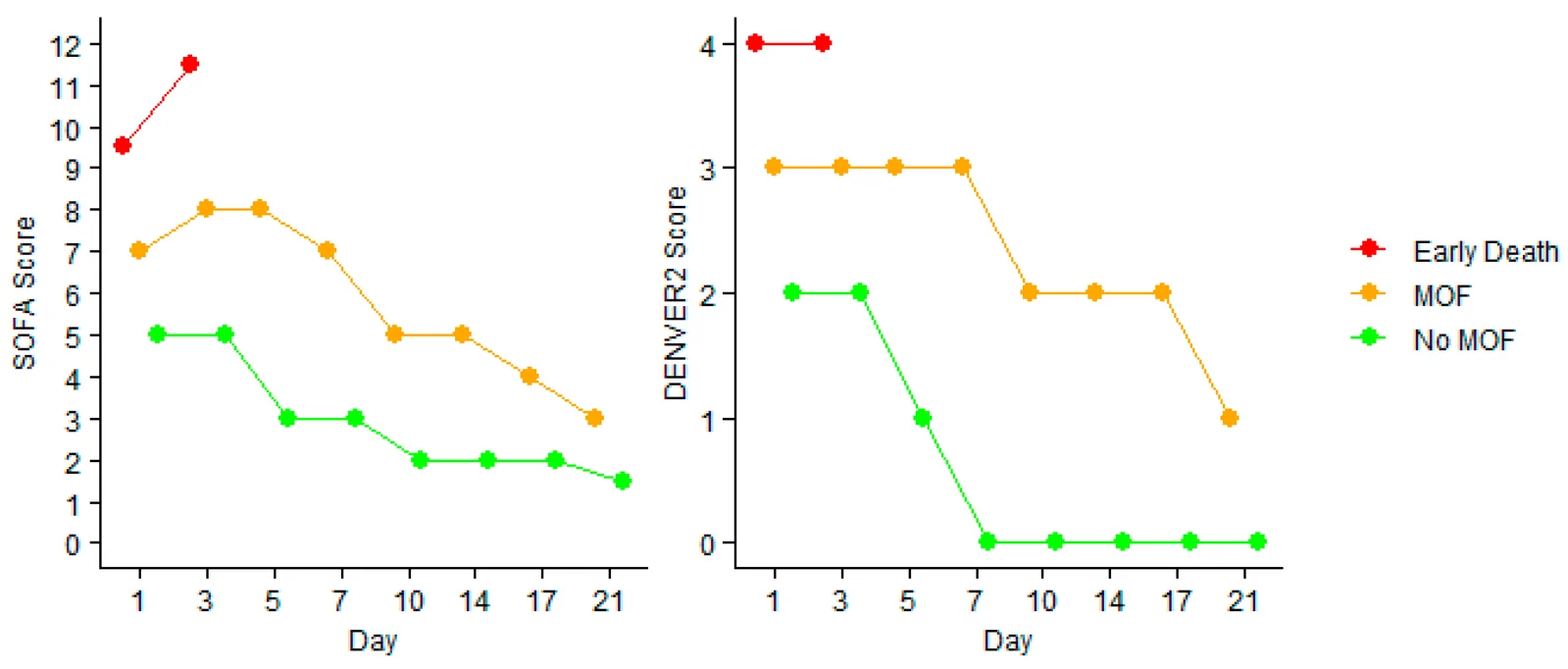
Longitudinal analysis of multi-organ dysfunction scores according to group (early death, MOF, no MOF) and day of intensive care unit admission. Points represent median values at each time point. SOFA = Sequential Organ Failure Assessment. MOF = multi-organ failure.

Figure 3 shows median SOFA and DENVER2 scores by day of admission, separated according to group. Patients in the early death group had the highest MODS scores on admission and at day 3. Patients who developed MOF had higher MODS scores than patients who did not develop MOF at every time point, including at days 1 and 3. Patients without MOF tended to return to normal physiology (DENVER2 score of 0) by day 7, whereas those who developed MOF continued to have elevated DENVER2 scores throughout the study period. The SOFA score remained persistently elevated throughout the study period in all groups.

### 3.3. Influence of Individual Organ Failures

**Figure 4:**
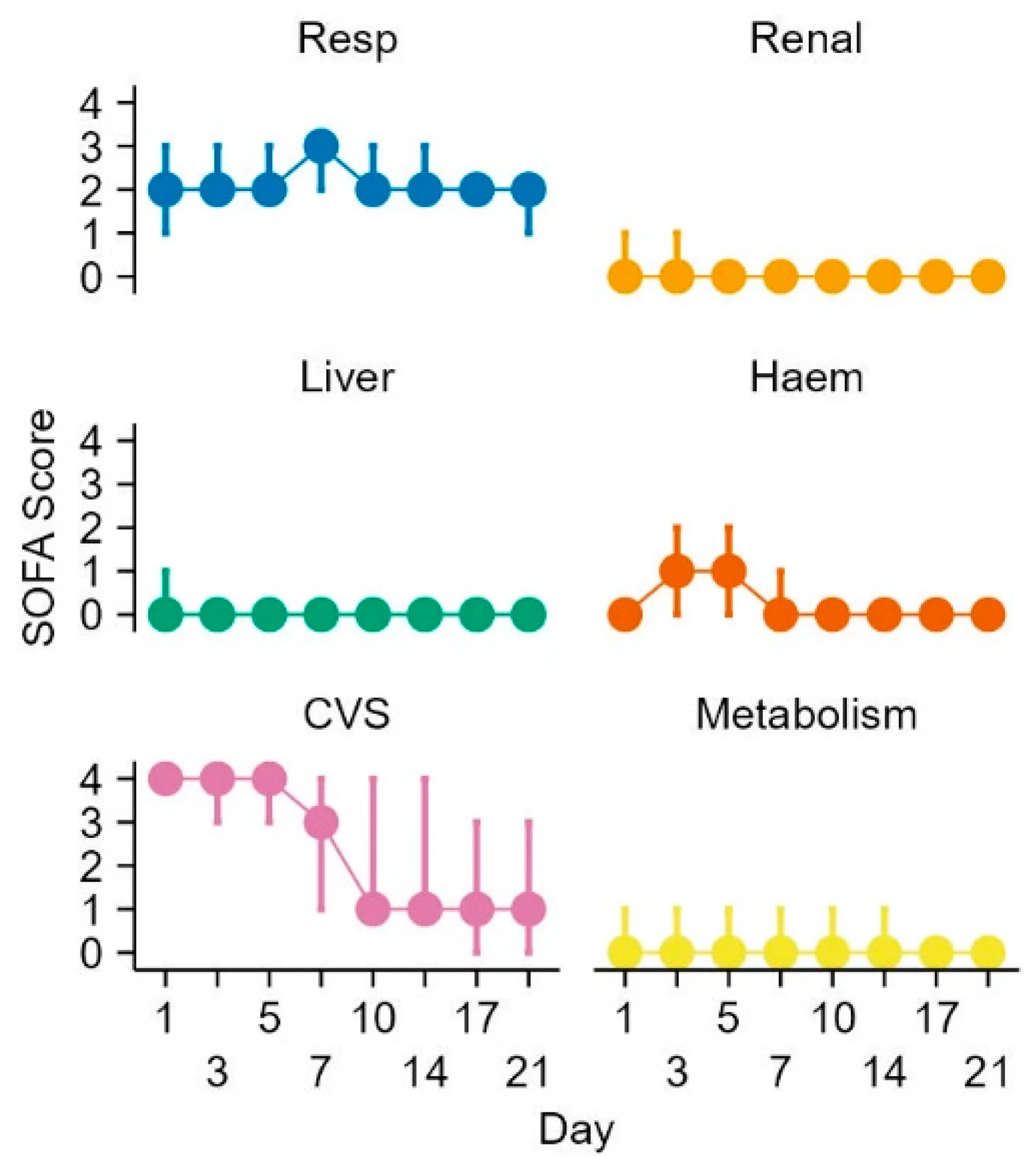
Time course of individual organ dysfunction according to day of intensive care unit admission. Points represent median values at that time-point and bars represent interquartile range. SOFA = Sequential Organ Failure Assessment. Resp = respiratory. Haem = haematological. CVS = cardiovascular system.

Figure 4 shows the median organ-specific SOFA score by day of admission to intensive care. Cardiovascular dysfunction peaks early at day 1 and remains severe until day 7, after which it falls precipitously. Median respiratory dysfunction score plateaus at 2 throughout the admission, with a small upwards blip to 3 around day 7. Haematological dysfunction was uncommon, peaking in severity between day 3 and day 7. Renal and liver dysfunction and insulin resistance were uncommon, with a median score of 0 at all time points.

**Figure 5:**
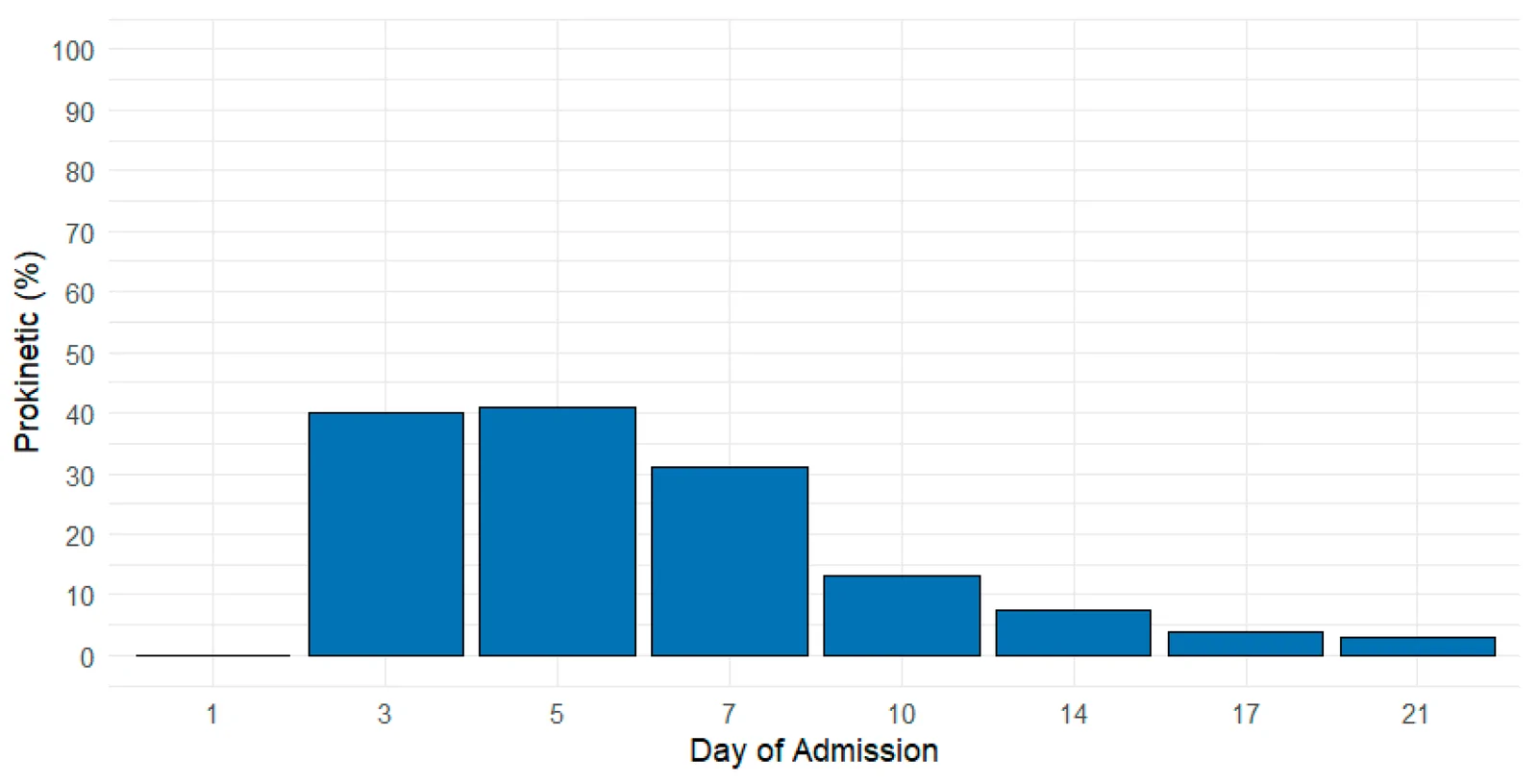
Prevalence of prokinetic medication use, a surrogate for feed intolerance and gut failure, according to day of admission to intensive care unit.

No patient required parenteral nutrition at any point in their ICU stay. As shown in Figure 5, the prevalence of prokinetic use (a surrogate for gut failure) peaked at 41% on day 5 before gradually declining to a low of 3% on day 21.

Table 2 shows the prevalence of individual organ failures after day 3, stratified according to survival to hospital discharge. Renal failure (i.e., creatinine > 300 umol/L or urine output < 500 mL/day) was associated with a lower chance of survival to hospital discharge. The requirement for CRRT was also associated with a reduced chance of survival to hospital discharge. There was no association between survival to hospital discharge and any other organ failure.

### 3.4. Risk Factors for Multi-Organ Failure During ICU Admission

Table 3 shows the results of multivariable logistic regression models for MOF during the early (<7 days) and late (>7 days) phases. During the early phase of ICU admission, TBSA, inhalational injury and presence of sepsis were independently associated with the occurrence of MOF. In the late phase of ICU admission, only sepsis and the Charlson comorbidity scale were independently associated with MOF. The association between sepsis and MOF was much stronger in the late phase than the early phase.

### 3.5. Initial Shock Severity and Risk of MOF

In univariable analyses, multiple markers of shock severity on admission to the ICU were associated with increased odds of MOF in the sub-set of 113 patients who survived the resuscitation period, including mean arterial blood pressure (MAP), heart rate, base excess, APACHE-II score and day 1 SOFA score (Table 4). MAP, base excess and day 1 SOFA score remained independently associated with MOF when accounting for age, TBSA and presence of inhalational injury.

Patients in the MOF group received higher volumes of intravenous fluids in the first 24 h of ICU admission than patients in the no MOF group, 3.78 (2.97–4.68) vs. 2.93 (1.04, 4.41) mL/kg/TBSA, *p* = 0.02. In multivariable logistic regression analysis, higher volume fluid resuscitation (measured in mL/kg) was associated with increased odds of MOF, even when accounting for age, TBSA, inhalation injury and APACHE II score (OR 1.02 (1.01–1.03) per each additional mL/kg fluid, *p* = 0.002).

**Figure A1:**
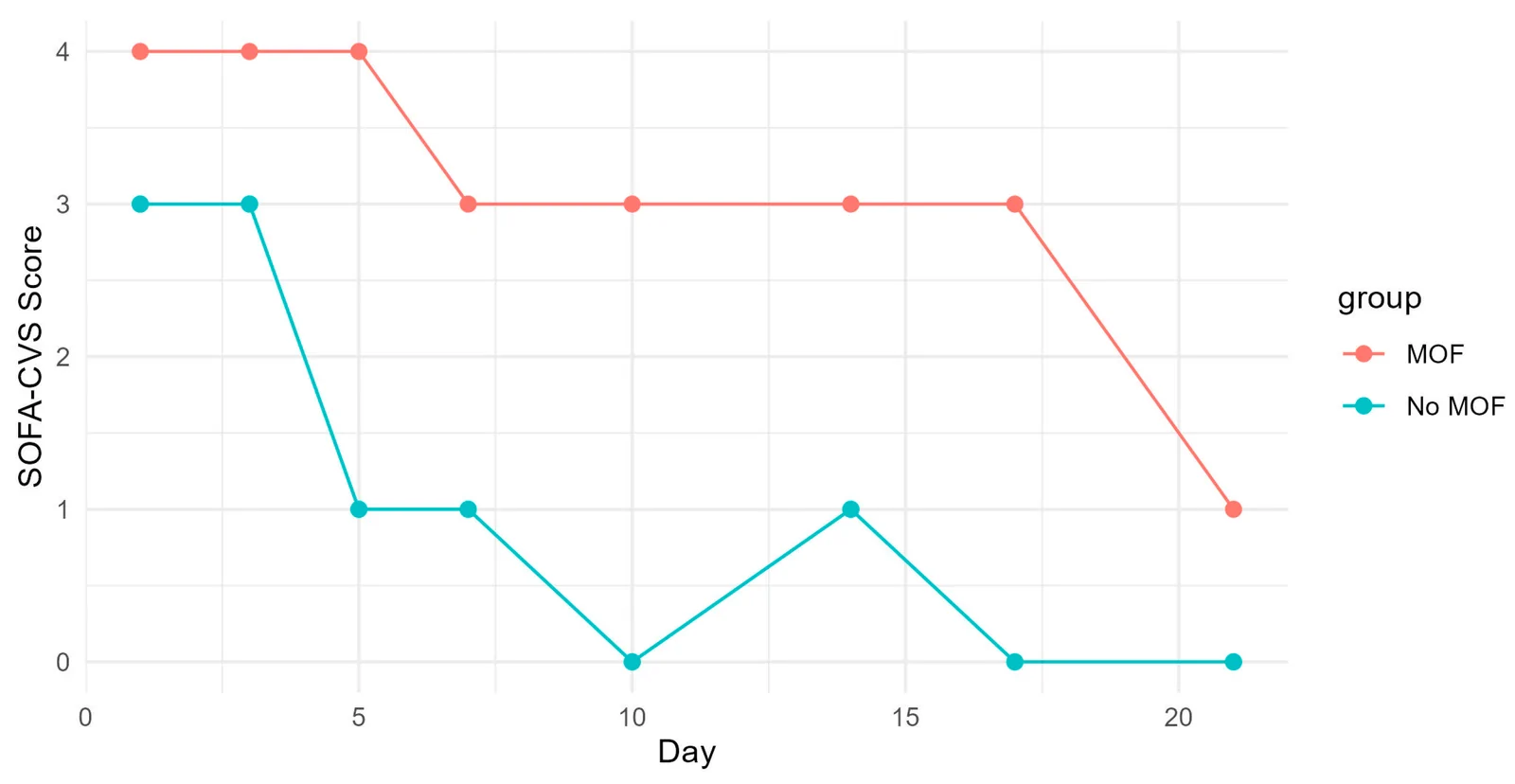
Median SOFA-CVS (Sequential Organ Failure Assessment Cardiovascular Sub-scale) score according to day of admission to intensive care and development of MOF (multi-organ failure). Higher scores indicate higher vasopressor requirement. For exact definitions, see [17].

Patients in the MOF group required higher doses of vasopressors on average than patients in the no MOF group, both during the resuscitation period (Appendix A, Table A1) and throughout their ICU stay (Appendix A, Figure A1).

## 4. Discussion

In our cohort of burn ICU patients spanning nearly a decade of admissions, we have closely detailed the onset and evolution of MOF and sepsis and presented three key findings. First, our data supports the concept of two distinct subtypes of burn multi-organ failure: sterile MOF, which occurs in the first week, and septic MOF, which occurs later. Following the resuscitation phase, there appears to be a bifurcation between day 7 and day 10 when patients either recover or develop persistent MOF, and this seems likely to be driven by infection. Second, we have demonstrated that renal failure is a highly significant marker of poor prognosis in the burns ICU. There is also a strong association between requirement for CRRT and mortality, although CRRT was commonly initiated for reasons other than overt renal failure. Third, we have shown that critical illness severity upon presentation to hospital and on admission to ICU are both associated with development of MOF.

In our cohort, early death occurred predominantly in the most elderly/frail with the most severe burns. This group of patients does not survive long enough to develop infection and instead succumbs to sterile multi-organ failure related to burn injury [12]. In an analysis of over 6000 deaths from burns in the United States, most deaths were associated with a rapid (<72 h) decline, as was also seen in our cohort [29]. In the same study, burn-shock was the cause of most deaths within the first week, with MOF and sepsis becoming most prevalent from week 3 onwards. In another cohort, patients who died without sepsis were found to be older and died much earlier, despite a smaller average burn size [1]. This could potentially be mediated by frailty, reflecting poor physiological resilience to the acute insult of a burn [30]. In our study, 61% of the early death group were considered frail, compared to 14–15% in the groups who survived the resuscitation phase, supporting this hypothesis. The difference in characteristics between groups may also reflect prognostic pessimism, as most deaths in the early death group were preceded by compassionate withdrawal of life-sustaining therapies.

The rate of MOF in our study was higher than reported by previous studies, peaking at 70% on day 5 [1,6,7,31]. This can be explained by the wide variation in criteria used to characterise MOF, as well as our selection of an ICU-specific cohort. Unsurprisingly, MOF was associated with worse outcomes in terms of total LOS, ICU LOS and ventilator days, in keeping with previous studies [1,6]. However, we did not demonstrate a statistically significant mortality detriment in patients who developed MOF. This contrasts with previous findings, including a large paediatric cohort study, which found a mortality of 87% in MOF compared to only 5% without [7]. In our study, mortality is numerically higher in the MOF group, and the lack of statistical significance could be simply explained by underpowering. However, it could also be explained by the fact that we excluded patients who died within 3 days from our comparison, as we considered these to be a clinically distinct cohort from those who developed MOF after completing resuscitation. A study from two adult burn centres in the US demonstrated improved long-term survival in patients with major burns compared to less severe burns in patients who survived to hospital discharge, despite a greater incidence of MOF and sepsis [32]. We may be seeing a similar phenomenon in our cohort; i.e., surviving long enough to develop MOF indicates an inherent physiological resilience that protects against mortality.

Like previous studies, we have reported an association of age, comorbidities, TBSA, inhalational injury and sepsis with MOF [33,34,35]. However, by dividing the timeline into early (<7 days) and late (>7 days), we were able to show the varying influences of these factors at different points throughout the ICU admission. During the early phase, organ failure is driven by the burn/inhalational injury itself, with a modest influence of sepsis, suggesting a persistence of the sterile multi-organ failure seen during the resuscitation phase. There is likely also an influence from comorbidity in this phase, as although only a trend was found in our study, strong associations have been reported in other cohorts [31,36]. In the late phase, compared to the early phase, there was almost a 7 times stronger association with sepsis (although the precision of this estimate was low). In fact, after 7 days, TBSA and inhalational injury were no longer significantly predictive of MOF. This shows the principal importance of infection and sepsis in the latter phases of burn ICU care. However, it is difficult to know the temporal relationship between sepsis and MOF. In a cohort of 175 severely burnt adult patients, 83% of episodes of MOF were preceded by infection, suggesting that infection was driving MOF [35]. In our study, MOF prevalence appeared to peak several days prior to the peak in sepsis prevalence, hinting at distinct aetiologies. This likely represents the two subtypes of burns-MOF, with sterile-MOF peaking early in admission and septic MOF peaking later. We observed a “bifurcation” between day 7 and day 10, where patients who never developed MOF normalised their physiology (median DENVER2 score of 0), but patients who developed MOF had persistent organ dysfunction. Given the high prevalence of sepsis at this time point, it is possible that the development of sepsis (or not) between days 7 and 10 is key. The underlying biological basis for this bifurcation warrants further investigation but may be related to inflammation-mediated immune exhaustion, or a tip in the balance between pro-inflammatory and immunosuppressant mediators and cytokines [37,38,39].

Renal failure was the only individual organ failure associated with mortality in our study. Despite an overall low rate of renal failure (9%), we identified a strong association with death in this group. There was also a mortality detriment associated with CRRT that was even more pronounced; however, this is complicated by the fact that CRRT is used for purposes unrelated to renal failure in our unit (e.g., temperature control). These findings are in keeping with meta-analyses of similar studies of ICU patients with burns, where the presence of acute kidney injury (AKI) was associated with an 11.3 (7.3–13.4) higher odds of mortality [40]. The low rate of renal failure in our study may be due to the relatively stringent diagnostic criteria we applied, as studies that have used alternative criteria have demonstrated higher rates of acute kidney injury (up to 60%) [41]. However, previous large cohort studies in both adults and children have demonstrated a similarly low rate of renal failure, with strong mortality implications when present [1,7]. Burns and sepsis have a shared pathophysiology involving systemic inflammation, microvascular dysfunction and capillary leak, all of which can drive kidney injury, and this may explain the poor prognosis associated with AKI in burns [42].

We report several other organ-specific findings worthy of discussion. First, cardiovascular failure, which was severe and almost ubiquitous in the first week of admission, was not associated with mortality. Similarly, respiratory failure was common throughout admission but did not represent a survival detriment. These findings, taken alongside previous studies, suggest that clinicians should avoid prognosticating or making withdrawal decisions based on the presence of shock or respiratory failure in these patients [1,7]. Liver failure was very rare, occurring in only one patient. Liver failure usually occurs later during burn MOF (after week 3) and so may not have been well captured in our study [6]. Haematological failure (i.e., severe thrombocytopenia) was also rare but occurred early in the admission between days 3 and 5, perhaps reflecting the effects of large-volume fluid resuscitation and inflammation-related platelet consumption [43,44]. There was no significant association between haematological failure and mortality in this study, but thrombocytopenia has been associated with both mortality and sepsis in a larger cohort of patients with burns from our centre [45]. We found that feed intolerance (defined as the requirement for prokinetic agents and used as a surrogate for gut failure) was relatively common, peaking at 41% on day 5 of ICU admission and then declining. The timing of feeding failure in our burn cohort is in keeping with the general ICU population, where it also peaks around day 5 and then steadily declines [46]. Early enteral nutrition reduces mortality in severe burns, and failure to meet nutritional requirements is associated with poorer outcomes in observational data [47,48]. However, we did not find an association between feed intolerance and mortality in our cohort. This may reflect the relative success of measures taken to improve nutrition, including prokinetic agents and early post-pyloric feeding—demonstrated by the fact that none of our patients required TPN despite the severity of their burns. However, although guidelines exist regarding prokinetic use, prescription remains clinician-dependent and is likely to lag the onset of gut dysfunction. Future studies should record more patient-centred markers of gut dysfunction and their relationship to outcomes.

Our findings demonstrate that events during the resuscitation phase of a burn injury are associated with risk of MOF later in the admission. Lower MAP and base excess upon presentation to hospital and higher day 1 SOFA scores were associated with increased risk of MOF, independently of burn severity. Interestingly, in contrast, base excess (used as a marker of resuscitation adequacy) at 24 h was not associated with MOF in a similar cohort of burn ICU patients [35]. This suggests a potentially important opportunity for early resuscitation, including by prehospital colleagues, optimising physiology in the hyper-acute phase of a burn injury to prevent later multi-organ sequelae. We have also reported an association between fluid volume received in the first 24 h of ICU admission and MOF, which was independent of age, TBSA, inhalational injury and APACHE-II. This should be interpreted with caution, as the average time between admission and ICU admission was 7 h, and we were unable to determine fluid volumes given prior to admission. However, the concept of “fluid creep” and its association with poor outcomes have been well defined in the literature, and our findings are consistent with this [49]. Future prospective studies should utilise randomisation to compare fluid regimes, limiting residual confounding from clinician-driven prescribing.

There are limitations to our study that readers should bear in mind when interpreting the results. Firstly, the retrospective design could render our findings vulnerable to selection and information bias, as is the case with all studies of this nature. Future prospective work, including that ongoing by our team, will be needed to confirm the findings. Whilst strict consensus-based criteria were applied for the diagnosis of sepsis, the subjective nature of the criteria and retrospective nature of the study leave this outcome open to bias [5]. In addition, the relatively small sample size means the study is underpowered to find small differences between groups and likely explains the broad confidence intervals of certain presented analyses. Secondly, the selection of patients admitted to the ICU, which was intended to enrich for our outcome of interest (MOF), limits the generalisability of the findings to the general burn population. Episodes of sepsis on the burn ward following discharge from the ICU would not have been captured in our data, although these are unlikely to have been severe if not requiring re-admission to the ICU. Finally, our study covers a period of nearly 10 years, during which both the surgical and intensive care management of severe burns have changed in ways that may influence the risk of MOF, for example, through earlier excision surgery and changes in fluid resuscitation strategies [50,51]. For these reasons, our study should be considered hypothesis-generating rather than providing causal associations.

## 5. Conclusions

This study generates three hypotheses that should be confirmed in future studies. First, risk factors for MOF in severe burns differ according to the time from admission, with MOF after the first week appearing to be predominantly sepsis-related. Second, renal failure is the individual organ failure most strongly associated with mortality, and clinicians should consider this in their clinical decision making. Finally, the adequacy of initial resuscitation—both under-resuscitation and over-resuscitation—is directly related to the later risk of MOF.

## Figures and Tables

**Table 1:** Summary of overall study characteristics and comparison between early death, multi-organ failure and no MOF groups. Bold *p*-values are statistically significant. LOS = length of stay. ICU = intensive care unit. BMI = Body Mass Index. TBSA = total body surface area. g/L = grams per litre. APACHE II = Acute Physiology and Chronic Health Evaluation II score. *p*-values are adjusted for multiple comparisons using Bonferroni correction.

Characteristic	Overall (N = 131)	Early Death (N = 18)	MOF (N = 78)	No MOF (N = 78)	*p*-Value
Sex (Male)	102 (78%)	15 (83%)	60 (77%)	27 (77%)	>0.9
Race					>0.9
White	111 (85%)	14 (78%)	66 (85%)	31 (89%)	
Black	4 (3.1%)	0 (0%)	3 (3.8%)	1 (2.9%)	
Asian	13 (9.9%)	4 (22%)	6 (7.7%)	3 (8.6%)	
Mixed	3 (2.3%)	0 (0%)	3 (3.8%)	0 (0%)	
Current smoker	28 (21%)	10 (56%)	13 (17%)	5 (14%)	**0.037**
Acute alcohol use	27 (21%)	3 (17%)	20 (26%)	4 (11%)	>0.9
BMI (Kg/m^2^)	25.2 (22.3, 29.7)	25.5 (22.1, 31.0)	25.5 (22.3, 30.8)	25.1 (22.3, 29.1)	>0.9
Diabetes	18 (14%)	6 (33%)	9 (12%)	3 (8.6%)	>0.9
Mechanism					>0.9
Flame	111 (85%)	17 (94%)	68 (87%)	26 (74%)	
Flash	12 (9.2%)	0 (0%)	6 (7.7%)	6 (17%)	
Scald	2 (1.5%)	0 (0%)	1 (1.3%)	0 (0%)	
Contact	1 (0.8%)	0 (0%)	1 (1.3%)	0 (0%)	
Chemical	1 (0.8%)	1 (5.6%)	0 (0%)	0 (0%)	
Electrical	4 (3.1%)	0 (0%)	2 (2.6%)	2 (5.7%)	
Revised Baux score	99 (74, 120)	121 (94, 132)	103 (86, 122)	72 (50, 105)	**<0.001**
%TBSA	39 (22, 53)	43 (25, 60)	40 (29, 60)	30 (18, 39)	0.06
%TBSA full thickness/deep dermal	20 (8, 40)	36 (17, 60)	29 (10, 40)	9 (5, 20)	**0.011**
Inhalational injury severity					**0.014**
None	48 (37%)	3 (17%)	23 (29%)	22 (63%)	
Mild	32 (24%)	3 (17%)	23 (29%)	6 (17%)	
Moderate	21 (16%)	2 (11%)	17 (22%)	2 (5.7%)	
Severe	30 (23%)	10 (56%)	15 (19%)	5 (14%)	
Head and/or neck burn	111 (85%)	14 (78%)	69 (88%)	28 (80%)	>0.9
Escharotomy	66 (50%)	10 (56%)	42 (54%)	14 (40%)	>0.9
Admission carboxyhaemoglobin (%)	2.0 (1.2, 3.3)	3.5 (2.0, 6.9)	2.0 (1.3, 3.3)	1.6 (1.2, 2.4)	**<0.001**
Day 1 albumin (g/L)	31 (24, 37)	23 (21, 28)	31 (24, 36)	36 (31, 41)	**0.011**
Total theatre trips	4.0 (2.0, 9.0)	1.0 (1.0, 1.0)	7.0 (4.0, 11.0)	3.0 (2.0, 5.0)	**<0.001**
Injury to ICU admission (hours)	7 (5, 8)	6 (5, 8)	7 (6, 7)	6 (4, 9)	0.8
Sepsis	80 (61%)	0 (0%)	67 (87%)	12 (34%)	**<0.001**
APACHE II	10 (7, 15)	17 (14, 19)	10 (7, 14)	9 (4, 11)	**<0.001**
Total LOS	39 (13, 74)	NA	56 (28, 105)	35 (16, 45)	**<0.001**
ICU LOS	14 (6, 27)	NA	16 (10, 22)	4 (1, 9)	**<0.001**
Ventilated days	10 (0, 22)	NA	9 (0, 17)	25 (16, 28)	**<0.001**
Ventilator-free days at 30 days	11 (0, 22)	NA	9 (0, 17)	25 (16, 28)	**<0.001**
Survival hospital discharge	88 (67%)	NA	59 (76%)	29 (83%)	0.4

**Table 2:** Association of individual organ failures with survival to hospital discharge. CRRT = continuous renal replacement therapy. Feed intolerance is defined as requirement for prokinetic agents after day 3. Insulin resistance is defined as requirement for intravenous insulin after day 3. Individual organ failures are defined as an individual SOFA score >/= 3. *p*-values are adjusted for multiple comparisons using Bonferroni correction. Bold *p*-values are statistically significant.

	Survived to Hospital Discharge		
Characteristic	Yes N = 88	No N = 25	*p*-Value
Cardiovascular Failure	65 (74%)	20 (80%)	>0.9
Respiratory Failure	50 (57%)	18 (72%)	>0.9
Renal Failure	5 (5.7%)	9 (36%)	**0.003**
CRRT	14 (16%)	16 (64%)	**<0.001**
Haematological Failure	3 (3.4%)	5 (20%)	0.1
Liver failure	1 (1.1%)	0 (0%)	>0.9
Insulin resistance	20 (23%)	12 (48%)	0.11
Feed intolerance	54 (61%)	10 (40%)	0.5

**Table 3:** Multivariable logistic regression models for MOF in the early (<7 days) and late phases (>7 days) of intensive care unit admission. CI = confidence interval. OR = odds ratio. TBSA = total body surface area burnt. Bold *p*-values are statistically significant.

Characteristic	OR	95% CI	*p*-Value
**MOF—Early**			
Charlson	1.29	0.99, 1.83	0.089
TBSA	1.08	1.03, 1.16	**0.007**
Inhalational Injury			
No	—	—	
Yes	5.56	1.39, 29.3	**0.023**
Sepsis			
No	—	—	
Yes	5.95	1.29, 43.7	**0.038**
**MOF—Late**			
Charlson	1.44	1.12, 1.92	**0.007**
TBSA	1.01	0.98, 1.03	0.5
Inhalational Injury			
No	—	—	
Yes	1.73	0.61, 5.07	0.3
Sepsis			
No	—	—	
Yes	42.2	12.1, 223	**<0.001**

**Table 4:** Logistic regression for MOF after day 3 according to markers of shock severity on admission. Multivariable models include age, total body surface area burnt and presence of inhalational injury as covariates. Bold *p*-values are statistically significant. MOF = multi-organ failure. BP = blood pressure. SOFA = Sequential Organ Failure Assessment. APACHE II = Acute Physiology and Chronic Health Evaluation score. OR = odds ratio. CI = confidence interval.

Characteristic	Univariable Association with MOF (OR, 95% CI)	*p*-Value	Multivariable Association with MOF (OR, 95% CI)	*p*-Value
Mean arterial BP	0.97 (0.95, 0.99)	**0.008**	0.98 (0.96, 1.0)	**0.039**
Heart Rate	1.03 (1.01, 1.05)	**0.005**	1.02 (1.0, 1.05)	0.057
Temperature	0.82 (0.61, 1.08)	0.2	0.96 (0.69, 1.33)	0.8
Base Excess	0.81 (0.71, 0.92)	**0.002**	0.87 (0.76, 0.99)	**0.042**
Lactate	1.14 (0.95, 1.39)	0.2	1.08 (0.89, 1.33)	0.5
Day 1 SOFA	1.71 (1.36, 2.23)	**<0.001**	1.56 (1.20, 2.12)	**0.002**
APACHE II	1.13 (1.03, 1.25)	**0.01**	1.07 (0.95, 1.22)	0.3

## Data Availability

The data are available upon request due to privacy concerns.

## Abbreviations

The following abbreviations are used in this manuscript:

UK	United Kingdom
TBSA	Total Body Surface Area
SOFA	Sequential Organ Failure Assessment
APACHE	Acute Physiology and Chronic Health Evaluation
ABA	American Burns Association
RRT	Renal Replacement Therapy
CRRT	Continuous Renal Replacement Therapy
ICU	Intensive Care Unit
BMI	Body Mass Index
MODS	Multi-Organ Dysfunction Syndrome
MOF	Multi-Organ Failure
R-Baux	Revised Baux score
LOS	Length of Stay
AKI	Acute Kidney Injury
OR	Odds Ratio
CI	Confidence Interval
CVS	Cardiovascular
CNS	Central Nervous System

## Appendix A

**Table A1:** DENVER 2 cardiovascular sub-scale (CVS) on day 1 and day 3, according to group. Higher scores indicate higher vasopressor requirement. For exact definitions, see [52].

Characteristic	Early Death	MOF	No MOF	*p*-Value
Day 1 DENVER2–CVS Score				<0.001
0	1 (5.6%)	2 (2.6%)	11 (31.4%)	
1	1 (5.6%)	5 (6.4%)	4 (11.4%)	
2	6 (33.3%)	54 (69.2%)	20 (57.1%)	
3	10 (55.5%)	17 (21.8%)	0 (0%)	
Day 3 DENVER2–CVS Score				<0.001
0	1 (17%)	5 (6.4%)	13 (37.1%)	
1	0 (0%)	6 (7.7%)	7 (20.0%)	
2	5 (83%)	50 (64.1%)	14 (40.0%)	
3	0 (0%)	17 (21.8%)	1 (2.9%)	
